# Role of *AdamTS-B* in *Drosophila* wing vein formation

**DOI:** 10.17912/micropub.biology.001680

**Published:** 2025-06-26

**Authors:** Jana Schulze, Uwe Töpfer

**Affiliations:** 1 Section of Molecular Hematology, Department of Hematology/Oncology University Hospital Freiburg, University of Freiburg, Freiburg im Breisgau, Baden-Wurttemberg, Germany; 2 School of Science, Technische Universität Dresden, Dresden, Saxony, Germany; 3 Department of Biology, Philipps University of Marburg, Marburg, Hesse, Germany

## Abstract

The development of *Drosophila* wing veins is a complex morphogenetic process that depends on the interplay of different signaling pathways, including EGFR, BMP, Notch, Hedgehog and Wnt. Basement membranes (BMs) and proteases that process BM components play a crucial role in controlling the morphogen spreading and associated patterning required for proper organ formation. Here we show, that *AdamTS-B* is required for the proper development of *Drosophila* wing veins. Knockdown of *AdamTS-B* results in various phenotypes, including additional veins, delta branches, and wandering veins within different longitudinal veins, though there are no differences in the cross veins.

**Figure 1. AdamTS-B is required for proper vein formation in the wing of D. melanogaster.**
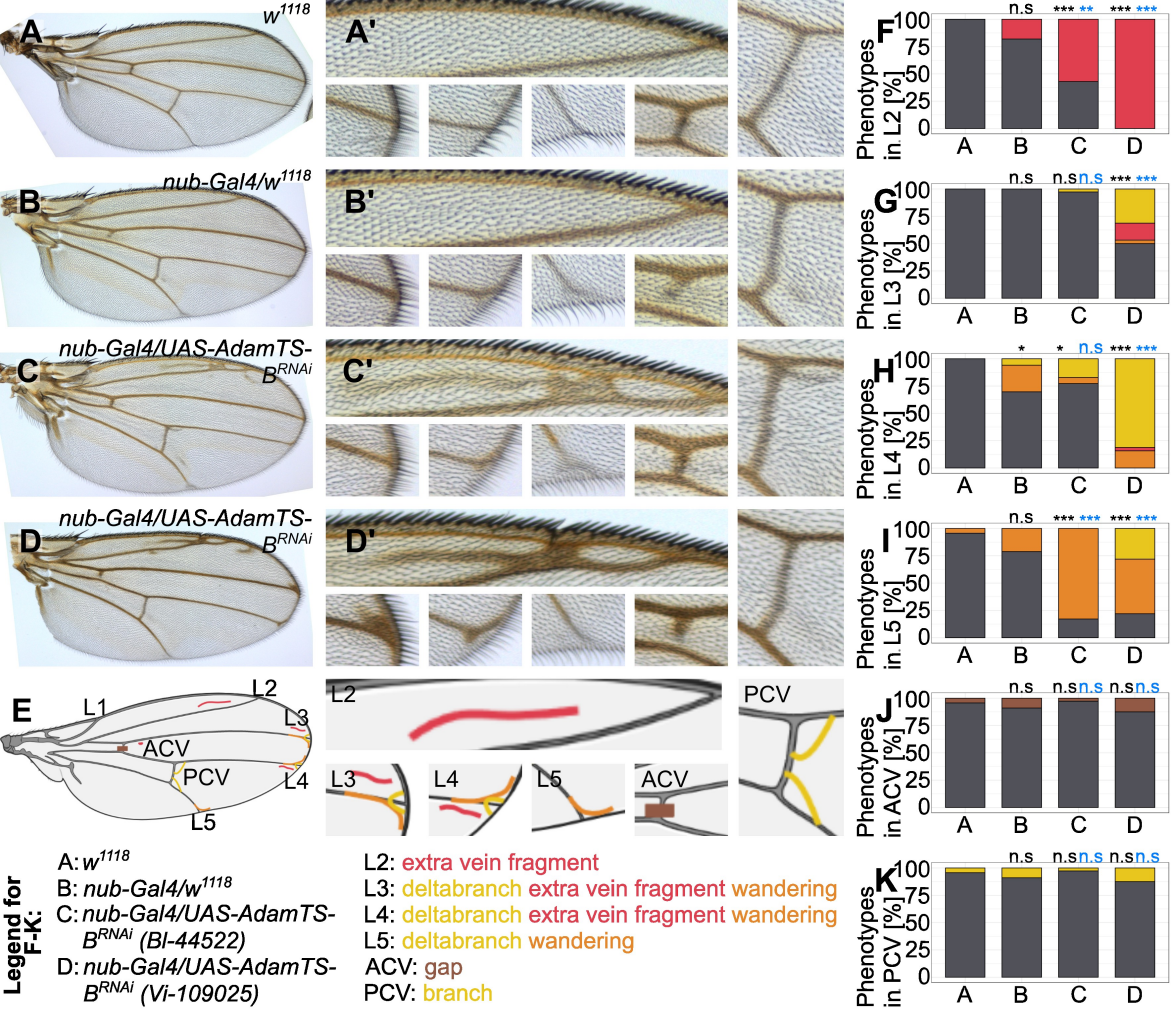
**(A-D')** Wings **(A, B, C, D)** and close-up of each vein region **(A', B', C', D')** are shown for the genotypes *w ^1118^* **(A, A')** and *nub-Gal4 /w ^1118^* **(B, B')** as controls, *nub-Gal4 /UAS- AdamTS-B ^RNAi^* (BDSC_44522) **(C, C')** and *nub-Gal4 /UAS- AdamTS-B ^RNAi^* (VDRC_109025) **(D, D')** . **(E)** Schematic of a wing showing the observed phenotypes. **(F-K)** Quantification of the observed phenotypes in each genotype for the veins L2 **(F)** , L3 **(G)** , L4 **(H)** , L5 **(I)** , ACV **(J)** and PCV **(K)** . The significance was tested in comparison with the *w ^1118^* (black asterisks) and *nub-Gal4 /w ^1118^* (blue asterisks) controls. Significances were estimated using Chi-square test. Significance levels: * = p < 0.05, ** = p < 0.01, *** = p < 0.001. n = 23 ( *w ^1118^* ), 33 ( *nub-Gal4 /w ^1118^* ), 35 ( *nub-Gal4 /UAS- AdamTS-B ^RNAi^* (BDSC_44522)), 32 ( *nub-Gal4 /UAS- AdamTS-B ^RNAi^* (VDRC_109025)).

## Description

The BM is a thin extracellular matrix that underlies epithelia and surrounds most tissues and organs, thereby mediating biophysical and biochemical cues for cells required for morphogenesis (Khalilgharibi & Mao, 2021; Sherwood, 2021; Töpfer, 2023). Those biochemical cues are mainly regulated by the ability of the BM to bind morphogens that are required for patterning (Patel et al., 2008; Wang et al., 2008). The remodeling of the BM by protease cleavage and subsequent degradation of the BM components is consequently an important step in the limitation of morphogens, patterning and morphogenesis. The *Drosophila* wing disc is composed of two opposing epithelial layers, which share a common BM that surrounds the organ. The formation of the *Drosophila* wing veins depends on the strict control of BMP morphogen distribution as well as the antagonism of Notch and EGF signaling, Hedgehog and Wnt pathways (Blair, 2007). The precise regulation of these patterning leads to a fully developed wing with five longitudinal veins (L1-L5) and two cross veins (anterior (ACV) and posterior (PCV)) ( Fig. 1A ). The AdamTS gene group is known to cleave extracellular matrix components and be important for cell migration and organ sculpting (Agarwal et al., 2022; Gonsior & Ismat, 2019; Ismat et al., 2013; Lhamo & Ismat, 2015; Skeath et al., 2017; Töpfer et al., 2024). *AdamTS-B* is one of four genes in *Drosophila* encoding a ‘A disintegrin and metalloprotease with thrombospondin motif' and an ortholog of human *AdamTS-7* and *AdamTS12* (Öztürk-Çolak et al., 2024). Knockdown of *AdamTS-B* has been shown to result in wing vein formation phenotypes. Butchar et al. 2012 identified *AdamTS-B* as a downstream gene of EGFR signaling and a knockdown with a ubiquitous Gal4 driver ( *Act5C-Gal4* ) led to an extra vein in L2. Based on this, Pham et al 2018 dissected the wing phenotypes of *AdamTS-B* using different driver lines and different UAS-dsRNA lines. In this study, we replicated an experiment form Butchar et al. 2012. and Pham et al. 2018 by knocking down *AdamTS-B ,* but with other genetic and environmental conditions.

For tissue-specific knockdown, we used *nubbin-Gal4 ( nub-Gal4 ),* which is active in the developing pouch region, the cells that give rise to the adult wing (Azpiazu & Morata, 2000). We analyzed the morphology of wings from *w ^1118^* (Fig.1A and A') and *nub-Gal4 /+* (Fig.1B and B') control flies as well as two different UAS-dsRNA lines for *AdamTS-B* knockdown *, nub-Gal4 /UAS- AdamTS-B ^RNAi^ ( BDSC_44522 )* (Fig.1C and C') and *nub-Gal4 /UAS- AdamTS-B ^RNAi^ ( VDRC_109025 )* (Fig.1D and D'). First, we quantified the presence of extra veins in L2 (Fig.1A-F). Knockdown of *AdamTS-B* with the BDSC_44522 (from here on BDSC line) and the VDRC_109025 (from here on VDRC line), which showed full penetrance of the phenotype, resulted in significant differences to *w ^1118^* as well as to *nub-Gal4 /+* controls (Fig.1F). In contrast to L2, in the L3-L5 regions, in addition to the extra vein phenotypes, we also observed the presence of delta branches and so-called wandering veins (illustrated in Fig.1E, Fig.1A-D' and G-I). In the L3 region, we observed the presence of all three phenotypes (extra vein, delta branch and wandering vein) with significant differences between the *AdamTS-B* knockdown with the VDRC line compared to both controls, while we found no differences for the BDSC line (Fig.1G). In L4, we found significant differences between the two controls with wandering veins and delta branches in *nub-Gal4 /+* controls (Fig.1H), as well as a significant difference between *w ^1118^* and the BDSC knockdown line. *AdamTS-B* knockdown with the VDRC line resulted in fully penetrance with all three possible phenotypes and significant differences compared to both controls (Fig.1H). In the last region for longitudinal veins, L5, both knockdown lines show significant differences compared to both controls, but only delta branches and wandering veins and no extra veins (Fig.1I). Finally, in the region of ACV and PCV, we found no differences in terms of the phenotypes just described and also presence of gaps, which are present in low penetrance in all genotypes in the AVC (Fig.1J).

In summary we can draw four conclusions. First, our results are consistent with the finding of Butchar et al. 2012 and Pham et al.2018, that knockdown of *AdamTS-B* can lead to extra veins in L2 (Fig.1F). Notably, while Pham et al. 2018 described extra veins in L2 with the *MS1069-Gal4* driver, they found a decreased percentage of extra veins in L2 with *AdamTS-B* knockdown using *nub-Gal4* , which is in contrast to our results. Second, in L3 and L4 the VDRC line shows various phenotypes, namely extra veins, delta branches and wandering veins, that are not significantly different between the *nub-Gal4* control and the BDSC line (Fig.1G and H). These observations have not been described previously. Third, in L5 we observe delta branches and wandering veins in both *AdamTS-B* knockdown conditions (Fig.1I). Wandering veins in L5 were also described in Pham et al. 2018. Fourth, the cross veins are not affected by *AdamTS-B* knockdown. To conclude, while our results mainly support the findings of Butchar et al. 2012 and Pham et al. 2018, on the one hand we show a result that is in contrast to Pham et al. 2018, specifically the knockdown of *AdamTS-B* led in our results to an increase of extra veins in L2 and in Pham et al. 2018 to a decrease. On the other hand, we also found a much higher penetrance of phenotypes in every longitudinal vein, at least with the VDRC line. One reason might be the difference regarding culturing conditions. In contrast to Pham et al. 2018, we kept our crosses at 29 °C, which might increase RNAi efficiency. In line with Pham et al. 2018 we observed differences regarding the different UAS lines. The VDRC line shows an overall much higher penetrance of phenotypes than the BDSC line. However, it is unclear whether the higher penetrance of the phenotypes in the VDRC line is due to a possible higher knockdown efficiency of this line or whether this line shows off-target effects in contrast to the BDSC.

*AdamTS-B* was identified as a target gene of EGFR pathway using microarray experiments, where ectopic activation of EGFR results in increased AdamTS-B expression (Butchar et al., 2012). The fact that reduction of *AdamTS-B* through RNAi led to the presence of extra veins (Butchar et al., 2012; Pham et al., 2018) and ectopic expression of *AdamTS-B* to the loss of veins (Pham et al., 2018) are reminiscent to the modulation of the EGFR signaling pathway (Brentrup et al., 2000; Sturtevant et al., 1993; Sturtevant & Bier, 1995). In addition, a genetic interaction analysis in which *AdamTS-B* knockdown phenotypes were attempted to be rescued revealed that genetic reduction of the EGFR ligands *kern* in combination with either *vein* or *spitz* reduces the proportion of wings with extra veins, suggesting a direct or indirect involvement of *AdamTS-B* in EGFR signaling at the ligand level (Butchar et al., 2012). Our results support the idea that *AdamTS-B* negatively regulates EGFR pathways, hence in our condition *AdamTS-B* knockdown shows stronger phenotypes in all longitudinal veins, which is evident since EGFR affects all longitudinal veins (Fig.1A-I). The exact mechanism by which *AdamTS-B* modulates the EGFR pathway and longitudinal vein formation is unknown. We propose a model in which *AdamTS-B* influences activation of EGFR signaling, controlling the morphogenesis of all five longitudinal veins, by remodeling the BM during larval stages when cell fate is determined by morphogen gradients (De Celis, 1998) and before the BM is degraded during wing growth and elongation (Diaz-de-la-Loza et al., 2018).

## Methods

***Drosophil* a genetics and husbandry**

The fly stocks used were *nub-Gal4* [Bloomington Drosophila Stock Center (BDSC), BDSC_25754], *w ^1118^* (BDSC_3605), *UAS- AdamTS-B ^dsRNA^* (BDSC_44522) and UAS- *UAS- AdamTS-B ^dsRNA^* [Vienna Drosophila Resource Center (VDRC), VDRC_109025]. Flies were kept at 25°C on standard food. Crosses were raised at 29°C.

**Wing dissection**

Flies were stunned with CO _2_ and the desired female flies were transferred to a block dish containing isopropanol. The wings were dissected from the body using forceps. The wings were transferred to tubes containing isopropanol using a cut pipette tip and stored until mounting in a mixture of isopropanol and Euparal (ratio 1:1).

**Wing quantification**

The pictures were taken using the bright field of the Observer.Z1 (air objective: 5x). For wings, that can't be captured with one photograph, two pictures were made and afterwards stitched together using ImageJ (Preibisch et al., 2009). Each wing was analysed for all veins.

**Statistical analysis**

To evaluate significances Chi-square testin R was used (R Core Team, 2021).

## Reagents

**Table:** 

***Drosophila* strains **	**Genotype**	**Identifier**		**Available from**
nub-Gal4	* P{w[+mC]=UAS-Dcr-2.D}1, w[1118]; P{w[+mW.hs]=GawB}nubbin-AC-62*	BDSC_25754	Bloomington Drosophila Stock Center	
w ^1118^	*w ^1118^*	BDSC_3605	Bloomington Drosophila Stock Center	
UAS-AdamTS ^dsRNA^	y[1] sc[*] v[1] sev[21]; P{y[+t7.7] v[+t1.8]=TRiP.HMC02914}attP2	BDSC_44522	Bloomington Drosophila Stock Center	
UAS-AdamTS ^dsRNA^	*P{KK114135}VIE-260B*	VDRC_109025	Vienna Drosophila Resource Center	
